# Progress and trends on machine learning in proteomics during 1997-2024: a bibliometric analysis

**DOI:** 10.3389/fmed.2025.1594442

**Published:** 2025-08-15

**Authors:** Chao Tan, Hao Liu, Zhen Zhang, Xinyu Liu, Yinquan Ai, Xiumin Wu, Enlin Jian, Yongyan Song, Jin Yang

**Affiliations:** Clinical Medical College & Affiliated Hospital & College of Basic Medicine, Chengdu University, Chengdu, China

**Keywords:** machine learning, proteomics, bibliometric, visual analytics, trend

## Abstract

**Objective:**

Despite growing interest in the application of machine learning (ML) in proteomics, a comprehensive and systematic mapping of this research domain has been lacking. This study addresses this gap by conducting the first large-scale bibliometric analysis focused exclusively on ML-driven proteomics, aiming to elucidate its knowledge structure, development trajectory, and emerging research trends.

**Methods:**

A total of 5,156 publications from the Web of Science Core Collection (1997–2024) were retrieved and analyzed. Bibliometric tools including CiteSpace 6.4.R1, VOSviewer 1.6.18, Scimago Graphica, and the R package bibliometrix were used to extract and visualize key bibliometric indicators. After data cleaning and de-duplication, analyses were conducted on keyword co-occurrence, citation networks, leading journals, influential authors, and institutional collaboration patterns to construct a comprehensive landscape of ML applications in proteomics.

**Results:**

The number of publications has grown exponentially since 2010, with an average annual growth rate of 12.53% and a notable surge of 65.14% occurring between 2019 and 2020. The United States emerged as the most productive country, while the Chinese Academy of Sciences led among institutions. AlphaFold2-related research received the highest citations, reflecting the transformative role of deep learning in protein structure prediction. Thematic clustering revealed key research foci, including deep learning algorithms, protein–protein interaction prediction, and integrative multi-omics analysis. The field is characterized by strong interdisciplinary convergence, involving computer science, molecular biology, and clinical research. High-impact journals and influential authors were also identified, providing benchmarks for academic influence and collaboration.

**Conclusion:**

This study offers the first comprehensive bibliometric analysis of ML in proteomics, revealing key themes such as deep learning, pretrained models, and multi-omics integration. Future efforts should focus on building interpretable models, enhancing cross-disciplinary collaboration, and ensuring secure, standardized data use to advance precision medicine.

**Systematic review registration:**

https://doi.org/10.17605/OSF.IO/F4WUG.

## 1 Introduction

Proteomics, the large-scale study of proteins, is a rapidly evolving field that plays a crucial role in understanding biological systems and disease mechanisms (1, 2). It involves the comprehensive analysis of protein expression, structure, function, and interactions within a given biological context. Proteomics has become indispensable in biomedical research, offering insights into disease pathology, biomarker discovery, and therapeutic target identification (3–5). Machine learning (ML) has indeed revolutionized the field of proteomics by providing innovative solutions to complex data analysis challenges. The integration of ML techniques with proteomics data has enabled significant advancements in protein identification, characterization, and the discovery of biomarkers for various diseases.

One of the applications of ML in proteomics is the enhancement of data analysis from mass spectrometry (MS) experiments (6). The development of algorithms that can process and analyze MS spectra more efficiently. These algorithms can assist in tasks such as peak picking, normalization, and missing data imputation, which are crucial for accurate data analysis. Supervised ML methods, such as random forests and support vector machines, have been employed to enhance the precision of these tasks, thereby facilitating biomarker detection and classification (7). Similarly, ML has been applied to spatial proteomics, where it aids in analyzing complex spatial data to understand protein localization and dynamics within cells (8). This interdisciplinary approach has provided insights into cellular processes and disease progression, demonstrating the potential of ML to enhance our understanding of cell biology and contribute to medical and drug discovery research (9). Furthermore, the application of ML in proteomics extends to the prediction of protein toxicity, where algorithms are used to analyze the properties and structural alerts of toxic proteins. This approach aids in understanding the mechanisms of protein toxicity and contributes to the development of peptide-based therapeutics (10). Additionally, ML has been utilized to predict missing proteomics values using transcriptomics and other biological features, enhancing the accuracy of protein quantification in proteomics studies (11).

Machine learning (ML) applied to proteomics for discovering and validating biomarkers may significantly change disease diagnosis, prognosis, and therapy. For instance, in the study of diabetic nephropathy, ML methods have been employed to screen urinary biomarkers, revealing proteins that are closely related to disease progression and could serve as potential diagnostic markers (12). Similarly, ML has been applied to high-dimensional proteomics datasets to identify biomarkers for Alzheimer’s disease, demonstrating the potential of these techniques in neurodegenerative disease diagnostics (13). In the realm of cancer research, ML has been utilized to connect histopathology imaging with proteomics data, particularly in kidney cancer (14). This approach has enabled the identification of diagnostic proteins that correlate with imaging-based predictions, providing novel insights into cancer biology and potential diagnostic applications. Furthermore, ML has been applied to differentiate interstitial lung disease by separating connective tissue disease-associated interstitial lung disease from idiopathic pulmonary fibrosis, showcasing its utility in respiratory disease diagnostics (15). The application of ML in proteomics is not limited to biomarker discovery. It also extends to the development of predictive models for disease diagnosis and prognosis. For example, in prostate cancer research, a ML pipeline has been developed to analyze clinical and proteomics data, resulting in the identification of peptides that could serve as biomarkers for early diagnosis (16). By integrating ML into various proteomics domains, researchers are better equipped to handle the challenges of big data and extract valuable biological insights, ultimately advancing the field toward more precise and effective therapeutic strategies.

Several reviews have summarized the advancements in the fields of ML and proteomics from multiple perspectives. In 2019, a review by Sonsare et al highlighted the progress in applying ML techniques to the analysis of proteomic data (17). A recent review provides insights into the progress, obstacles, and future opportunities for ML in the field of proteomics, particularly focused on the ML incorporated into proteomics tools and biomarker studies (18). A review by Neely et al focused on evaluating and exploring ML tools for realistic data modeling from multidimensional MS-based proteomics analysis of various samples or organisms (19). Another review offers guidance and suggestions for interdisciplinary experts planning to use ML techniques in multi-omics research (20). Even though ML is widely used in proteomics, there is still a lack of comprehensive systematic reviews assessing this interdisciplinary integration.

Bibliometric analysis has become a valuable method for comprehending the dynamics of research areas, providing insights into publication patterns, key studies, and collaborative networks (21). This method employs quantitative techniques to evaluate scientific literature, providing a comprehensive overview of a field’s development and identifying emerging areas of interest. Bibliometric analysis provides a data-driven perspective that can uncover patterns and trends in scholarly communication. In addition, it aids researchers in rapidly comprehending the progress and leading edges of a specific field of study. So far, there hasn’t been a bibliometric analysis conducted on the fields of ML and proteomics. Aiming to fill this gap, this bibliometric analysis offers a comprehensive and high-level summary of the present status of ML and proteomics, highlighting its potential to revolutionize biological understanding and change medical practices.

## 2 Materials and methods

### 2.1 Data sources and search strategies

The Web of Science Core Collection (WoSCC) is widely used as a primary database for publications involving ML methods. It is recognized as one of the most authoritative, comprehensive, and academically influential citation databases, offering extensive coverage across disciplines. Recognized as one of the most authoritative, comprehensive, and academically influential citation databases, WoSCC provides extensive data coverage. Publications within this database are retrieved using the Topic Search (TS) method, which integrates subject terms from the Mesh vocabulary to enhance search precision (22). This study utilized the WoSCC database to identify relevant publications based on the following search strategy: (TS = (((((((((((((((Machine learning) OR (Naive Bayes)) OR (Decision trees)) OR (Random Forest)) OR (Support vector machines)) OR (Gradient boosting decision tree)) OR (Adaptive boosting)) OR (Extreme gradient boosting)) OR (Light gradient boosting machine)) OR (Categorical boosting)) OR (Generalized additive model)) OR (Artificial neural networks)) OR (Data Mining)) OR (Deep learning)) OR (Transfer Learning)) AND TS = (((((((Proteome) OR (proteomes)) OR (proteomic)) OR (proteomics)) OR (proteomical)) OR (Peptidomics)) OR (protonically)))) and language = (English) and article type = (articles or reviews) and time span = (January 1997 to December 2024), Incomplete or unpublished literature was excluded, and 5156 results were included. The titles, authors, abstracts, keywords, and cited references were then imported into the scientific knowledge graph analysis tools.

### 2.2 Data analysis and visualization

The study utilized CiteSpace 6.4.R1, VOSviewer 1.6.18, Scimago Graphica, and the R package “bibliometrix” to conduct a comprehensive bibliometric analysis, with results presented through various forms of visual mapping. Following the initial retrieval of records from the WoSCC database, a rigorous data cleaning process was performed. This included standardizing bibliographic information and systematically identifying and removing duplicate entries based on unique identifiers.

## 3 Results

### 3.1 Analysis of annual publications and citations

**FIGURE 1:**
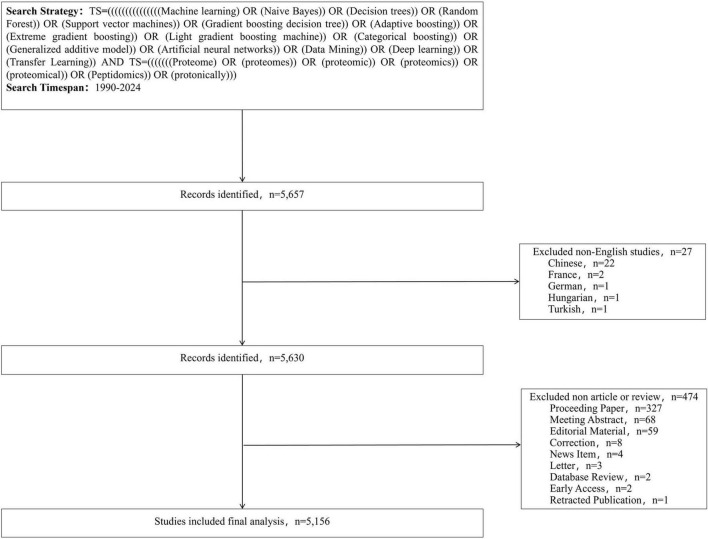
Flowchart for the research’s search process.

Based on the selection criteria, English-language articles published between 1997 and 2024 were included in this study, as illustrated in Figure 1.

**FIGURE 2:**
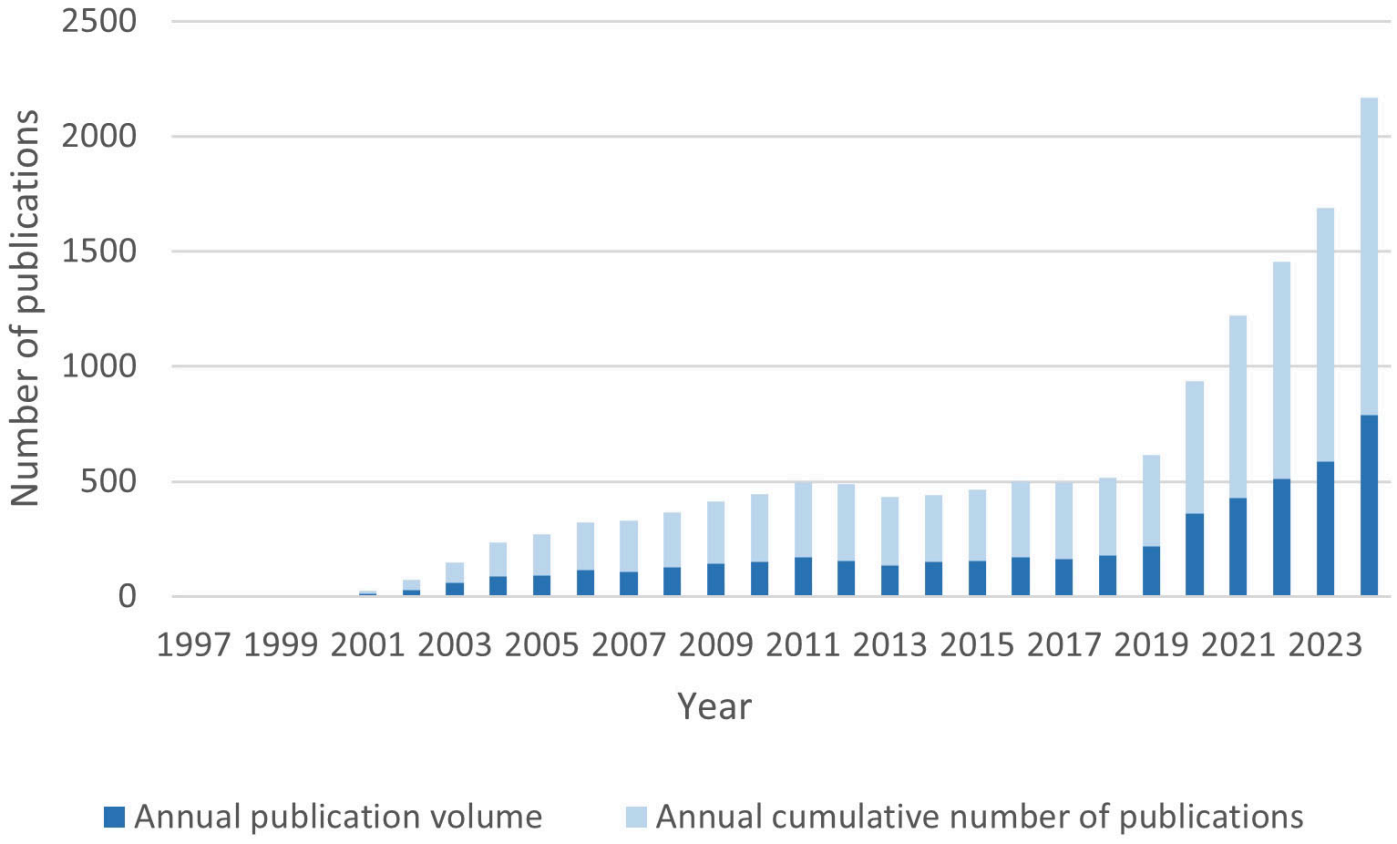
Analysis of annual publications and Annual cumulative publications.

According to our search conducted on December 31, 2024, a total of 5,156 publications were retrieved from the WoSCC database, comprising 4,440 research articles and 716 review papers. As shown in Figure 2, the number of publications related to proteomics and ML has exhibited a sustained and exponential growth trend from 1997 to 2024. Prior to 2010, the annual publication output in this field was relatively low, with fewer than 100 articles per year, totaling 423 articles over 13 years, averaging 32.5 articles per year, while citation counts were negligible. However, after 2010, the field experienced a notable surge, with annual publications rising from hundreds to thousands, marking the beginning of a decade of rapid expansion. The number of publications has grown exponentially since 2010, with an average annual growth rate of 12.53% and a notable surge of 65.14% occurring between 2019 and 2020. The year with the fewest publications was 1998, with only one article, whereas the most prolific year was 2024, with 790 publications. According to the latest growth trend estimation, the number of publications in 2025 is expected to exceed 1,000. This sharp upward trend highlights the rapid expansion of interdisciplinary research in proteomics and ML and underscores its increasing academic influence in recent years.

### 3.2 Analysis of countries/regions

This study provides a bibliometric analysis of the global research landscape on the application of ML in proteomics, examining publication output, academic influence, and international collaborations. Table 1 presents the top 10 contributing countries/regions, detailing their publication volume, centrality score, single-country publications (SCP), multi-country publications (MCP), and multi-country publications percentage (MCP%).

**TABLE 1:** Ten most productive countries and regions.

Country	Articles	Centrality	SCP	MCP	MCP%	H-index
USA	1289	0.19	997	292	22.7	136
China	1106	0.02	889	217	19.6	80
Germany	321	0.16	179	142	44.2	77
United Kingdom	298	0.24	149	149	50	73
India	189	0.08	139	50	26.5	34
Canada	180	0.05	115	65	36.1	56
Italy	150	0.08	118	32	21.3	44
Spain	137	0.1	80	57	41.6	44
Australia	123	0.13	60	63	51.2	45
France	110	0.14	59	51	46.4	45

SCP, single-country publications; MCP, multi-country publications; MCP%, multi-country publications percentage.

In the global research landscape of proteomics and ML, the United States, China, the United Kingdom, and Germany stand out as the dominant research forces, each demonstrating unique strengths in publication output, academic impact, and global collaboration. The United States leads with 1,289 publications and holds the highest H-index (136), and its centrality score (0.19) further underscores its critical role in the international collaboration network. However, its MCP% (22.7%) is relatively low.

China, ranking second with 1,106 publications, has exhibited remarkable growth in research output in recent years. However, its H-index (80) remains lower than that of the United States, and its centrality score is only 0.02. The United Kingdom, in contrast, serves as a pivotal hub in the global collaboration network. Despite its relatively lower publication count (298 articles), it holds the highest centrality score (0.24) and an MCP% of 50.0%, showcasing strong cross-border research partnerships. Germany is Europe’s most influential research center, with 321 publications, an H-index of 77, a centrality score of 0.16, and an MCP% of 44.2%.

Beyond these leading contributors, Canada, France, and Australia also exert significant influence in the field. Although their publication volumes are relatively lower, their H-index suggest strong research quality. Canada (H-index = 56, centrality = 0.05, MCP% = 36.1%) maintains moderate international collaboration, whereas Australia has the highest MCP%, highlighting its strong reliance on global partnerships. France, Spain, and Italy also play notable roles in international collaboration, each with an H-index above 44, underscoring their substantial research contributions. Meanwhile, India, despite having a considerable number of publications (189 articles), has a lower H-index (34) and an MCP% of 26.5%.

**FIGURE 3:**
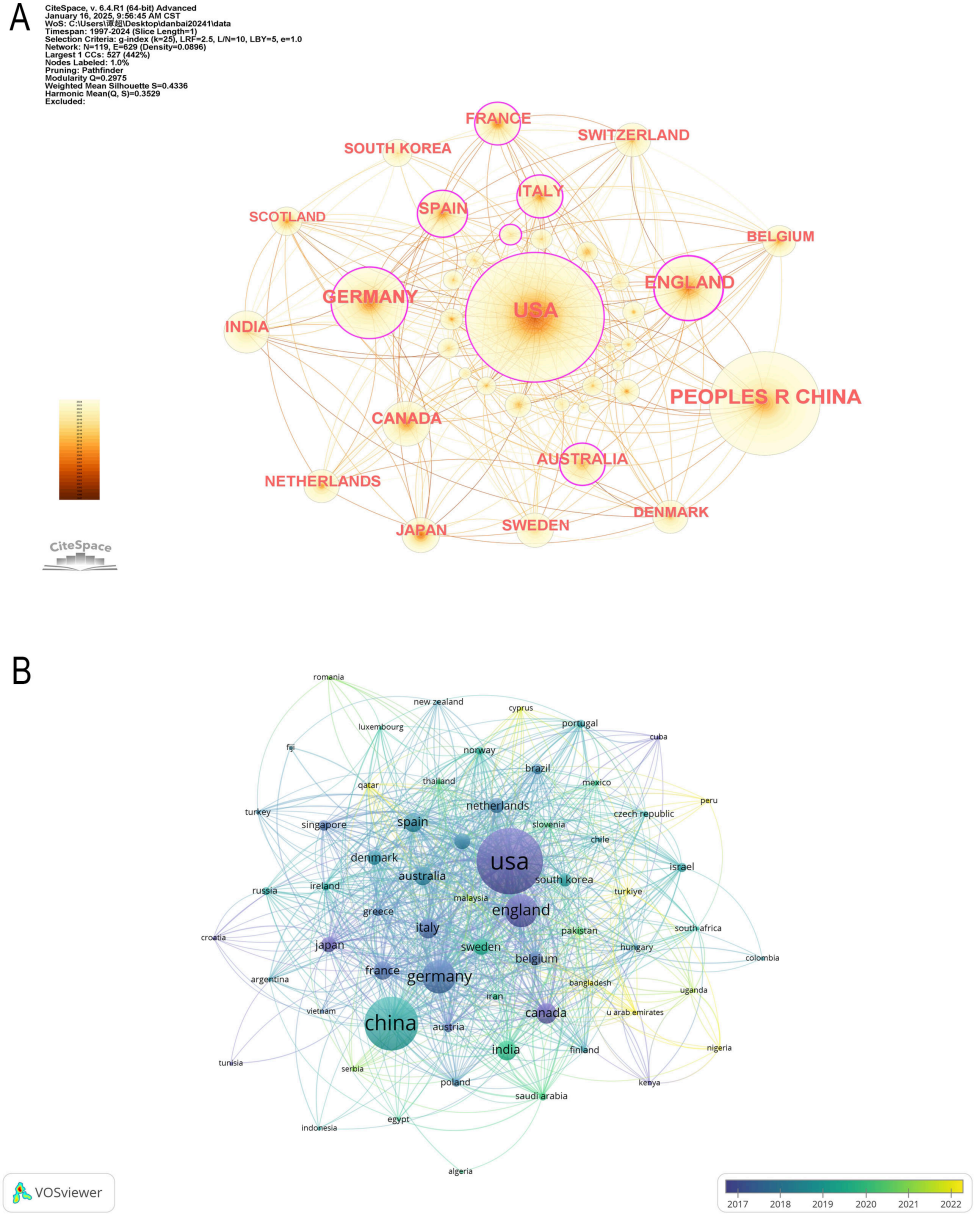
Collaboration of countries/regions in the field of machine learning and proteomics. **(A)** The countries/regions’ network visualization map based on CiteSpace. **(B)** Analysis of collaborative network visualization of countries/regions in VOSviewer.

**FIGURE 4:**
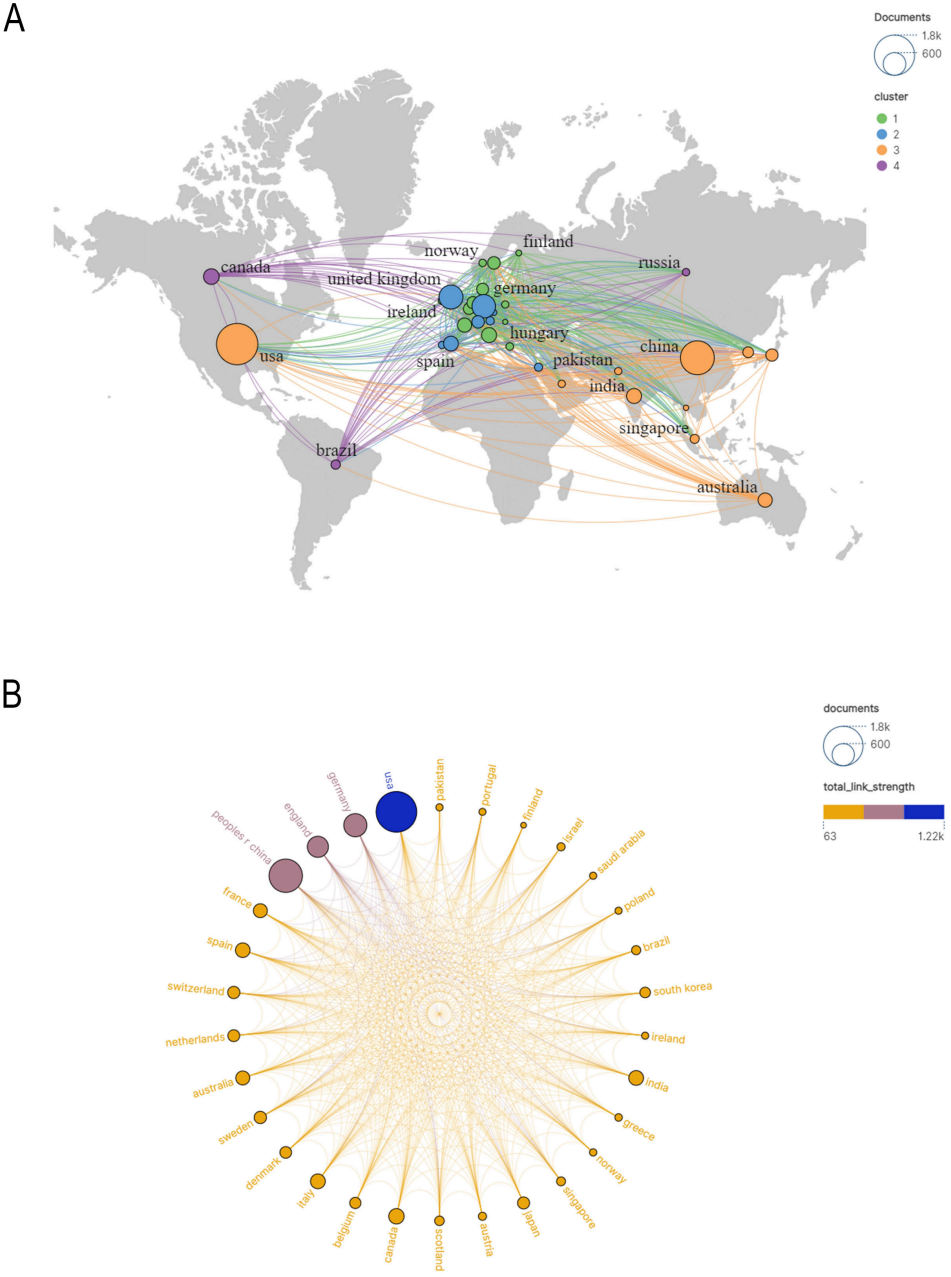
**(A)** Geographical map of publications. **(B)** Countries/regions involved in proteomics research using machine learning methods, the links between countries/regions indicate their collaborations and connections.

In Figure 3A illustrates the collaboration network among the top 18 countries, where network connections represent partnerships, and node sizes indicate publication volume. Figure 3B further visualizes publication volume and the timeline of collaborations. The United States, United Kingdom, Canada, and Japan emerged as early contributors, whereas the United Kingdom and Turkey have demonstrated significant research contributions in recent years. By integrating global data into a world map (Figure 4A), a global collaboration network was generated, revealing four distinct research clusters, with the United States exhibiting the most extensive collaborations, followed by Germany, the United Kingdom, and China. The color distribution and network connections emphasize a cluster-based collaboration structure rather than a uniformly distributed global cooperation network. Additionally, (Figure 4B) presents a chord diagram of collaborations among the top 30 countries.

### 3.3 Analysis of affiliations

**FIGURE 5:**
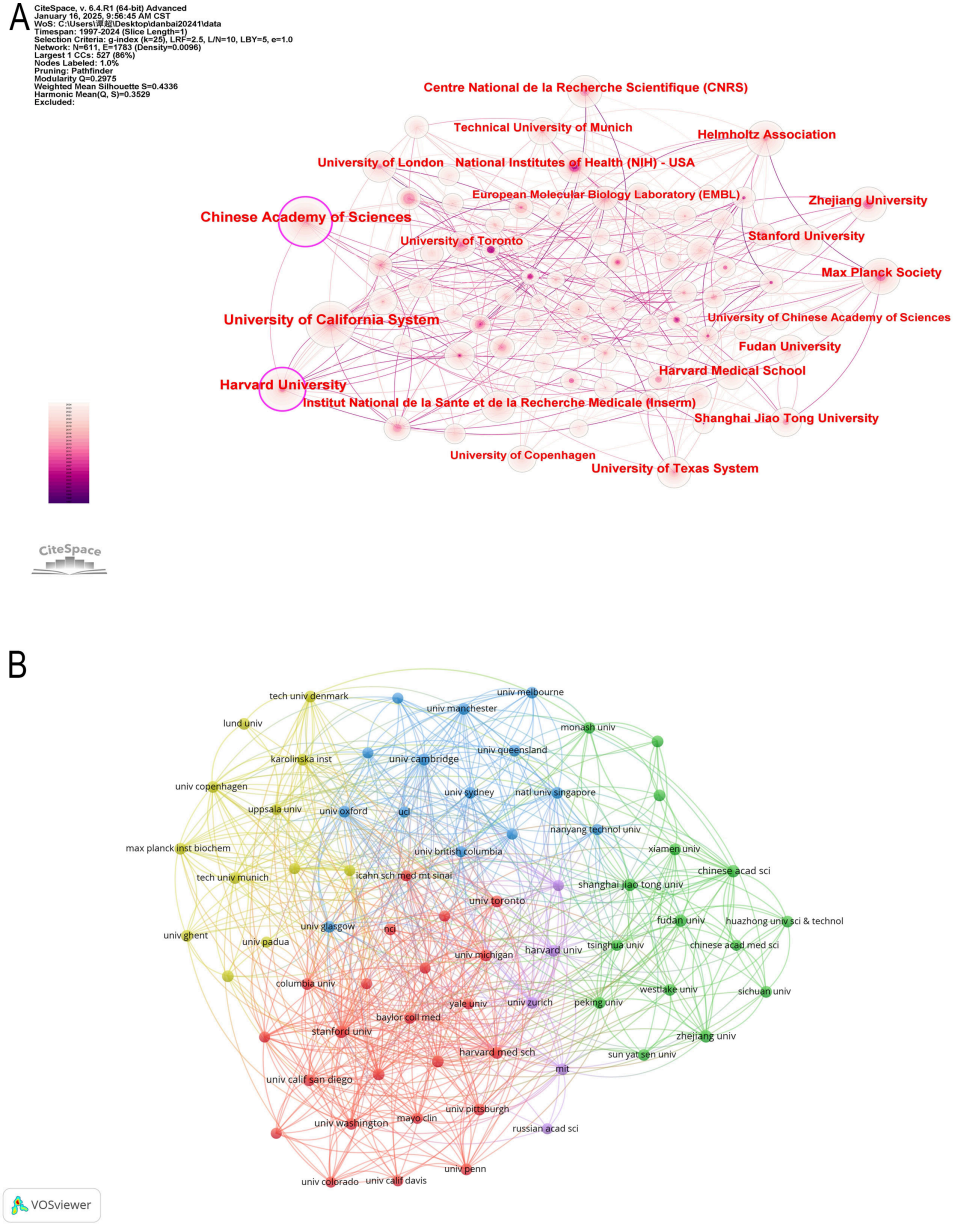
**(A)** Network map of institution analysis based on CiteSpace. **(B)** The institutions’ collaboration network visualization maps based on VOSviewer.

The institutional landscape of proteomics and ML research is shaped by a few dominant institutions that drive scientific advancements through extensive publications, strong academic influence, and global collaborations. Table 2 presents detailed information on the top 10 contributing institutions. The Chinese Academy of Sciences (CAS) leads in publication volume with 196 articles, followed by the University of California System (188) and Harvard University (168). However, research quality and citation impact, as reflected by the H-index, indicate that the University of California System (H-index: 51) and Harvard University (H-index: 47) exert a more substantial influence compared to CAS (H-index: 42). Notably, the Max Planck Society in Germany, despite producing only 96 publications, boasts the highest average citation per article (139.32), signifying its high research impact. Centrality, which measures institutional influence in global collaborations, highlights CAS (0.17), Harvard University (0.15), and the University of California System (0.12) as the most well-connected institutions in international research networks. While American institutions, including the University of California System, Harvard University, the University of Texas System, and NIH, dominate the field in both volume and impact, European institutions such as the Max Planck Society and CNRS (France) stand out for their high-impact research despite lower publication counts. Conversely, China’s Zhejiang University (93 publications, H-index: 22) is emerging as a key player, though its relatively lower citation impact suggests a need for greater international engagement. Figures 5A, B reveal a densely connected network, where the University of California System and Harvard University exhibit extensive global collaborations, whereas CAS, despite its high output, has a relatively weaker collaboration intensity. European institutions maintain strong intra-regional partnerships but have comparatively lower engagement with Asian research centers.

**TABLE 2:** The top 10 institutions including the volume of publications, average per item, H-index and centrality.

Rank	Organization	Centrality	Country	Documents	H-index	Average per item
1	Chinese Academy of Sciences	0.17	China	196	42	30.74
2	University of California System	0.12	American	188	51	96.93
3	Harvard University	0.15	American	168	47	68.6
4	University of Texas System	0.07	American	105	34	52.46
5	Harvard Medical School	0.02	American	103	37	71.19
6	Max Planck Society	0.06	Germany	96	45	139.32
7	Helmholtz Association	0.04	Germany	94	33	51.16
8	Zhejiang University	0.04	China	93	22	15.94
9	Centre National de la Recherche Scientifique (CNRS)	0.04	France	91	27	31.76
10	National Institutes of Health (NIH)-USA	0.12	American	90	35	75.74

As shown in Supplementary Table 1, funding in this domain is predominantly led by agencies based in the United States and China. The United States Department of Health and Human Services and the National Institutes of Health (NIH) top the list, supporting 819 and 816 publications respectively, underscoring the United States government’s strong investment in biomedical and computational research. China’s National Natural Science Foundation (NSFC) ranks third with 689 funded publications. The European Union (EU), UK Research and Innovation (UKRI), and Germany’s DFG also appear prominently, demonstrating substantial European engagement.

### 3.4 Analysis of authors

In recent years, the intersection of proteomics and ML has attracted significant scholarly attention and achieved remarkable progress. Table 3 lists the top ten most influential authors in this field along with their bibliometric indicators. Among them, eight scholars have published more than ten papers, demonstrating their high research productivity. Additionally, three scholars from China (Guo Tiannan, You Zhu-Hong, and Zheng Shu) reflect the country’s rapid development in this field, consistent with the findings in Table 1. Matthias Mann and Kuo-Chen Chou stand out with 7449 and 6770 total citations, respectively, with an average of 341.5 and 234.52 citations per paper, indicating their broad recognition in the academic community. In contrast, Harald Mischak, despite having the highest number of publications (38 papers), has a relatively lower H-index (19), suggesting that his influence relies more on high publication volume rather than a few highly impactful papers.

**TABLE 3:** Top 10 lead authors in the field of research.

Rank	Author	Country	H-index	Average per item	Documents	Citations
1	Mischak, Harald	Austrian	19	42.79	38	1232
2	Chou, Kuo-Chen	American	30	234.52	27	6770
3	Mann, Matthias	Germany	17	341.5	25	7449
4	Martens, Lennart	Belgium	17	31.28	24	715
5	Song, Jiangning	Australia	13	43.75	21	948
6	Guo, Tiannan	China	11	49.39	19	154
7	You, Zhu-Hong	China	15	42.68	17	738
8	Gonzalez-Diaz, Humberto	Spain	16	60.58	16	1059
9	Degroeve, Sven	Belgium	9	31.29	14	435
10	Zheng, Shu	China	9	21.13	14	288

**FIGURE 6:**
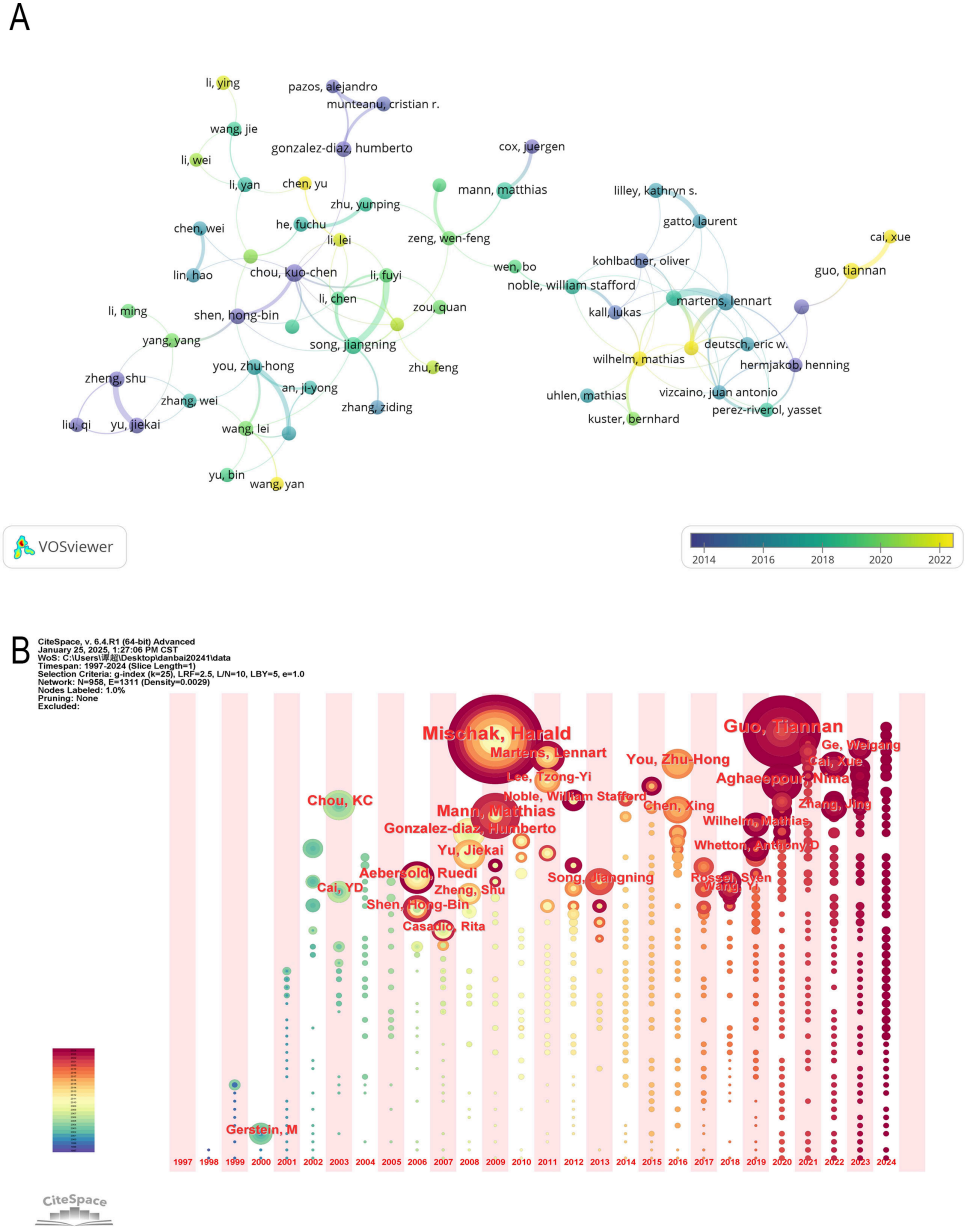
**(A)** Network map of author analysis based on VOSviewer. **(B)** Timezone of co-author.

We utilized VOSviewer to analyze the collaboration network (Figure 6A) and further examined the time-based distribution of these authors’ contributions using CiteSpace’s timezone view analysis (Figure 6B). This visualization spans the research period from 1997 to 2024, illustrating how scholars’ contributions have evolved over time.

Mischak, Harald is a prominent scholar in the field of proteomics and ML, with his research primarily focused between 2005 and 2015. His work has significantly impacted protein biomarker discovery, mass spectrometry data analysis, and the application of ML in proteomics (23–26). Similarly, Matthias Mann and Kuo-Chen Chou laid the foundation for this field during the same period, advancing proteomic data processing, ML algorithm optimization, and protein function prediction. Although their contributions were made earlier, their research continues to be widely cited today (27–29).

Between 2015 and 2022, You Zhu-Hong and Song Jiangning were highly active in the field, making significant contributions to PPI prediction, protein sequence classification, and the application of deep learning in proteomics, which provided a strong foundation for further advancements (30–36).

In recent years, Guo Tiannan (2020–2024) has emerged as a leading researcher in this domain, making notable progress in single-cell proteomics, deep learning applications in protein structure prediction, and the integration of multi-omics data for disease diagnosis, thus driving cutting-edge developments in the field (37, 38).

### 3.5 Analysis of journals

**FIGURE 7:**
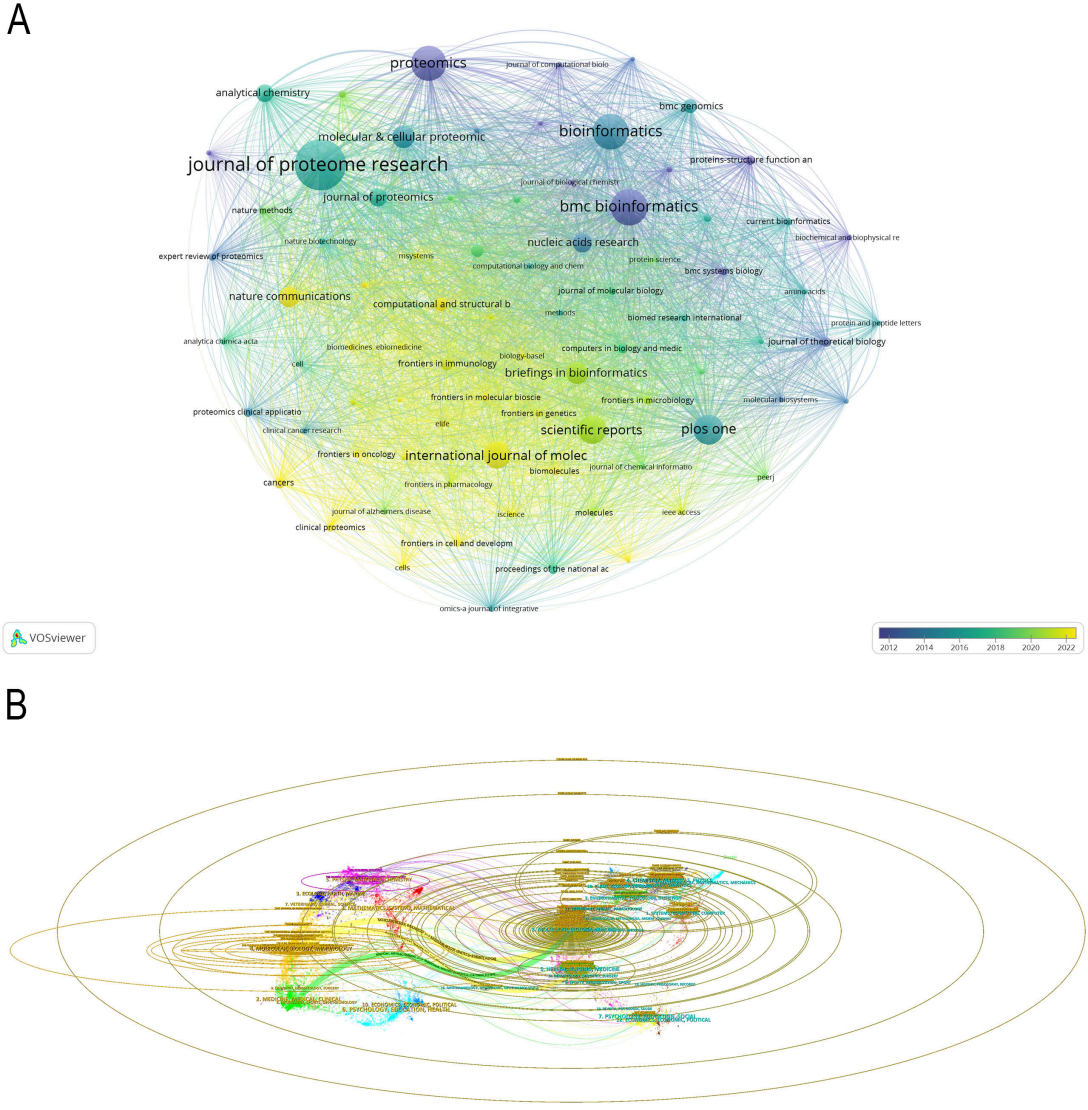
**(A)** Visual analysis of journals collaboration network in VOSviewer. **(B)** The dual-map overlay of journals. The labels on the left represent citing journals, the labels on the right represent cited journal, and colored paths indicate citation relationships.

This interdisciplinary field encompasses 1402 journals, of which 81 were selected for VOSviewer visualization analysis, with a minimum criterion of 10 publications per journal. The nuanced variation in color within the visualization effectively integrates the journal co-occurrence network with the publication timeline (Figure 7A). The CiteSpace-based scientific domain co-occurrence analysis (Figure 7B) illustrates the multidisciplinary integration trend of ML in proteomics research. The colors represent different disciplines: yellow/green areas are primarily related to molecular biology, genetics, and medicine, blue/purple areas correspond to mathematics, computer science, and statistics, while red areas reflect chemistry, physics, and materials science, indicating the deep intersection between proteomics and ML. The central region highlights the integration of proteomics and AI, mainly within medicine and bioinformatics, while the peripheral regions encompass mass spectrometry analysis, plant genomics, and precision medicine.

Additionally, we conducted an in-depth analysis of the top fourteen institutions with the highest publication volume (Table 4). Journal of Proteome Research has published 247 papers, making it the most prolific journal in this field and solidifying its central role in proteomics research. Bioinformatics, with 154 papers and an impact factor of 13.4, holds significant influence in computational biology and bioinformatics. Nucleic Acids Research, publishing 66 papers with the highest impact factor of 19.16, has received extensive citations, demonstrating its strong academic recognition and impact.

**TABLE 4:** Top 14 most cited journals.

Full journal name	Count	Country	IF
Journal of Proteome Research	247	United States of America	4.4
BMC Bioinformatics (BioMed Central Bioinformatics)	159	United Kingdom	2.9
Bioinformatics	154	United Kingdom	13.4
Proteomics	150	Germany	4.1
PLOS ONE (Public Library of Science ONE)	123	United States of America	3.7
Scientific Reports	113	United Kingdom	4
International Journal of Molecular Sciences	106	Switzerland	5.6
Molecular and Cellular Proteomics	84	United States of America	7.4
Briefings in Bioinformatics	82	United Kingdom	13.994
Nature Communications	75	United Kingdom	17.7
Nucleic Acids Research	66	United Kingdom	19.16
Journal of Proteomics	63	Netherlands	3.9
Analytical Chemistry	60	United States of America	8
BMC Genomics (BioMed Central Genomics)	43	United Kingdom	4.4

IF, impact factor.

In terms of publication distribution, seven of the top ten journals in this field originate from the United Kingdom, with a primary focus on bioinformatics and computational biology. The United States plays a leading role in experimental proteomics and data analysis, as reflected in the contributions of Journal of Proteome Research and Molecular and Cellular Proteomics. Meanwhile, journals from Germany, Netherlands, and Switzerland emphasize mass spectrometry, structural biology, and protein function prediction, providing crucial support for advancements in experimental methodologies and computational techniques.

### 3.6 Analysis of co-cited references

**FIGURE 8:**
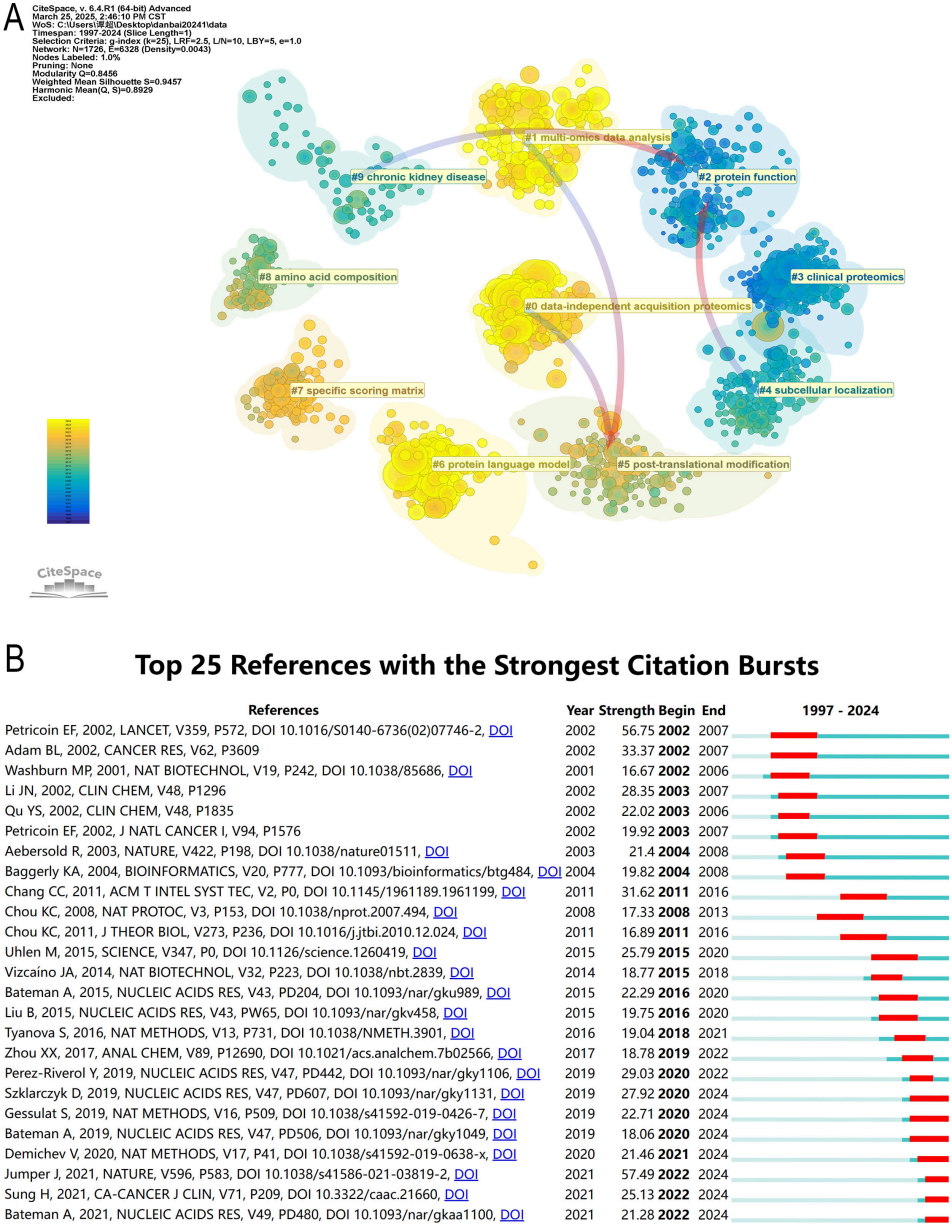
**(A)** Clustering of references based on similarity between references. The clustering is based on the degree of association between the literature and was divided into mainly 10 categories in different colors. **(B)** The top 25 references with the strongest citation bursts.

Co-citation analysis provides an objective and data-driven approach to understanding the foundational research and evolutionary trends within a scientific field (Figure 8A). ML in proteomics encompasses multiple key research areas, covering data processing, functional prediction, structural analysis, and clinical applications. Notably, the focal points shifted over time, significant topics had transitioned toward multi-omics data analysis, data-independent acquisition proteomics. Among these, data-independent acquisition proteomics (#0) is the most central and largest research area, where data-independent acquisition mass spectrometry is widely used for protein quantification, and ML plays a crucial role in data-independent acquisition data processing and protein identification. At the same time, multi-omics data analysis (#1) is advancing rapidly, with ML facilitating the integration of proteomics, genomics, transcriptomics, and metabolomics, driving progress in systems biology and precision medicine.

In Protein function (#2) research, AI and computational modeling are widely applied to PPI prediction, enzyme activity analysis, and sequence-based functional annotation. Protein language models (#6), such as AlphaFold and ESMFold, utilize deep learning Transformer architectures, significantly improving protein structure prediction and functional annotation accuracy. Additionally, Specific scoring matrix (#7) serves as the computational foundation for protein sequence alignment and similarity assessment, playing a crucial role in phylogenetic analysis and homology modeling.

Subcellular localization (#4) research helps predict the distribution of proteins within different cellular environments and their biological functions, while PTMs (#5) (e.g., phosphorylation, glycosylation, and ubiquitination) are essential for cell signaling and disease progression. Amino acid composition (#8) explores the relationship between protein amino acid sequences and their functional properties, with applications in protein design, stability prediction, and functional annotation. Furthermore, ML has extended its applications to (#3) Clinical proteomics, revolutionizing biomarker discovery, disease diagnosis, and personalized medicine, particularly in chronic kidney disease (#9), where proteomics-based approaches are used to identify key biomarkers, aiding in disease progression prediction and precision treatment.

To determine the most frequently cited studies in recent years, we conducted a comprehensive analysis of the top 25 most-cited references (Figure 8B). The publication year represents the time of the study, while the intensity of the citation burst reflects the level of attention these studies have received from the academic community. The start and end points indicate the duration for which these papers have been frequently cited, corresponding to the prominent red sections in the figure.

As of December 31, 2024, several studies continue to experience significant citation bursts, underscoring their pivotal contributions to proteomics, computational biology, and AI applications. Among them, the study by Szklarczyk D (2019, Intensity: 27.92, Time span: 2020–2024) introduced STRING v11, a comprehensive protein-protein association database that supports large-scale experimental data analysis and functional discovery. The sustained high citation rate suggests that STRING remains an indispensable tool for proteomics research and AI-driven network analysis (39). Similarly, Gessulat S (2019, Intensity: 22.71, Time span: 2020–2024) focused on deep learning applications in proteomics, particularly in spectral prediction models based on AI. The citation surge highlights the growing impact of AI in protein identification, mass spectrometry data interpretation, and proteomic analysis (9, 19, 40, 41). Bateman A (2019, Intensity: 18.06, Time span: 2020–2024) contributed to the development of protein databases, particularly in functional annotation and sequence classification (42). The continuous increase in citations reflects the expanding role of ML in large-scale protein function prediction, reinforcing the importance of automated protein classification. Demichev V (2020, Intensity: 21.46, Time span: 2021–2024) introduced DIA-NN, a neural network-based software for Data-Independent Acquisition (DIA) proteomics analysis (43, 44). The persistently high citation rate indicates that AI-enhanced DIA mass spectrometry is now widely applied in quantitative proteomics research, significantly improving the depth and precision of proteomic data analysis. Jumper J (2021, Intensity: 57.49, Time span: 2022–2024) authored the most highly cited study in this dataset, introducing AlphaFold2, developed by DeepMind, which revolutionized protein structure prediction. The exceptional citation surge reflects AlphaFold’s transformative impact on computational biology, structural proteomics, and pharmaceutical research, firmly establishing AI as an essential tool in structural biology (45). Sung H (2021, Intensity: 25.13, Time span: 2022–2024) published a study in CA: A Cancer Journal for Clinicians, providing global cancer statistics widely used for epidemiological research and clinical decision-making. The study further highlights the crucial role of cancer burden research in biomedical studies and public health policy formulation (46). Lastly, Bateman A (2021, Intensity: 21.28, Time span: 2022–2024) advanced protein annotation databases, emphasizing AI-driven automated classification of protein families, reinforcing the growing demand for accurate, large-scale protein classification tools and demonstrating the increasing integration of AI into bioinformatics and proteomics research (42).

This citation burst analysis is further supported by the co-citation network visualization shown in Supplementary Figure 1, which illustrates the intellectual structure and research frontiers within this domain. In the network, recent high-impact studies such as Jumper J (2021), Szklarczyk D (2019), Demichev V (2020), and Gessulat S (2019) occupy central and densely connected positions, marked by large yellow nodes that indicate both high citation frequency and recent influence. Their prominent locations and extensive linkages reflect their foundational roles in advancing protein structure prediction, protein-protein interaction analysis, deep learning-based spectral prediction, and AI-assisted quantitative proteomics. Moreover, purple-rimmed nodes such as Chang CC (2011) and Perez-Riverol Y (2019) represent key transitional works with high betweenness centrality, suggesting their influence in bridging diverse research themes in computational tools and proteomic data repositories. The temporal evolution shown through color gradients reveals a clear shift from early mass spectrometry-focused studies (Petricoin EF, 2002) to modern AI-driven approaches, highlighting the dynamic progression of the field.

### 3.7 Analysis of keyword

**FIGURE 9:**
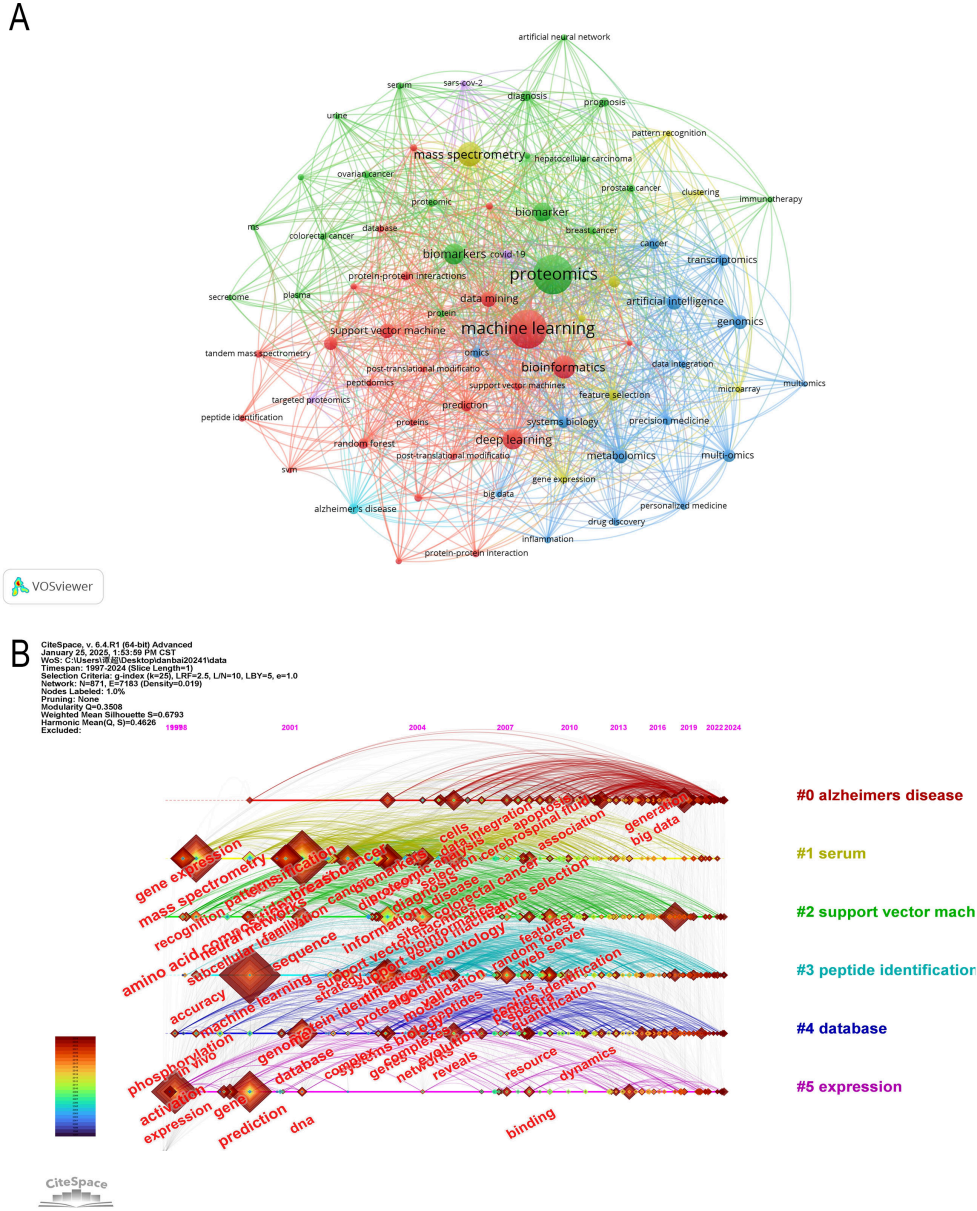
**(A)** Visual analysis of keywords collaboration network, different colors represent different clusters, and the size of nodes indicates their frequency. **(B)** The timeline graph of keywords in CiteSpace. Each horizontal line represents a cluster. Nodes size reflects co-citation frequency, and the links between nodes indicate co-citation relationships. Nodes occurrence year is the time when they were first co-cited.

In modern scientific research, interdisciplinary collaboration has become increasingly common, and keyword analysis can reveal the intersections between different disciplines. It can also help identify current research hotspots in the academic field and highlight emerging areas or technologies (47). The central position of proteomics and ML in the network reflects their core role in research, surrounded by numerous close connections with other keywords (Figure 9A). Terms such as “breast cancer,” “ovarian cancer,” “prostate cancer,” and “colorectal cancer” suggest that cancer research is a significant area within proteomics. ML may be applied to cancer diagnosis, prognosis, and personalized treatment strategies. Terms like “artificial intelligence,” “bioinformatics,” and “data integration” appear around proteomics, reflecting the trend of integrating AI models into bioinformatics workflows. These models are being used to enhance the efficiency of protein function prediction, biomarker identification, and proteomic data analysis. Nodes around “deep learning” and “data mining” represent an increase in the application of advanced computational methods in handling complex proteomic data. These methods are becoming central to large-scale data analysis, improving the ability to identify proteins, analyze PTMs, and explore PPI networks.

**FIGURE 10:**
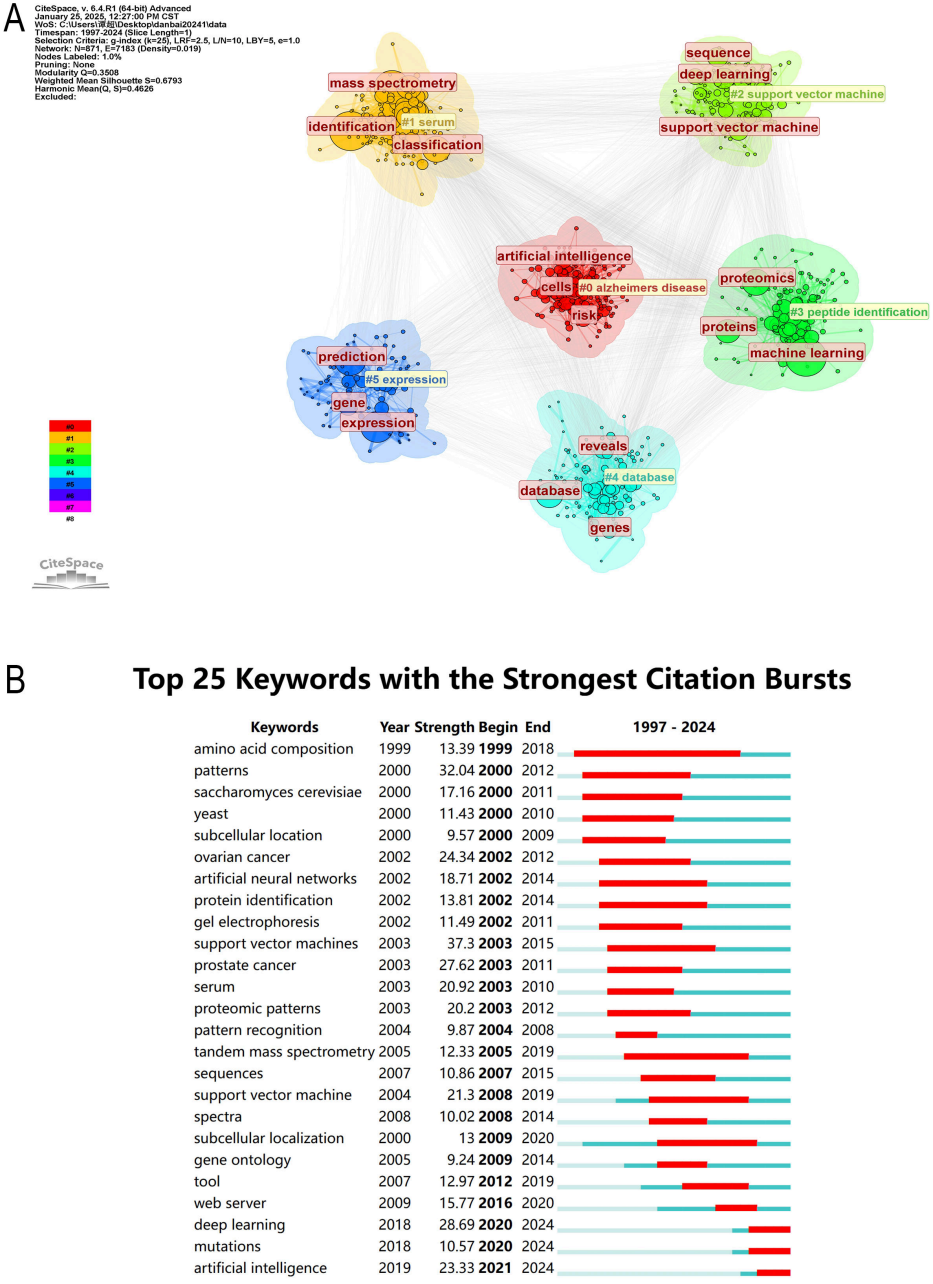
**(A)** Network map of the top 10 keyword cluster analyses based on CiteSpace. **(B)** Top 25 keywords with the strongest citation bursts (1997–2024).

The keywords were clustered using CiteSpace (Figures 9B, 10A) revealing 6 key clusters, Alzheimer’s Disease (#0), Serum (#1), Support Vector Machine (#2), Peptide Identification (#3), Database (#4), Gene Expression (#5). Supplementary Figure 2 further illustrates the thematic evolution of machine learning in proteomics across four time periods. In 1997–2005, early themes centered on mass spectrometry and biomarker discovery. From 2005 to 2015, focus shifted toward protein identification, bioinformatics, and feature selection. During 2015–2022, themes such as deep learning, proteogenomic, and data integration became prominent. Most recently, between 2022–2024, emerging keywords include AlphaFold, protein structure prediction, automation, and AI-based annotation, reflecting the ongoing expansion of AI applications in proteomics.

### 3.8 Analysis of keyword burst

The analysis of keyword burst maps helps identify research hotspots in a specific field over a certain period. Figure 10B presents the top 25 most frequently cited keywords, with the year indicating when each keyword first appeared and the strength reflecting its prominence. A higher strength value suggests a more significant occurrence of the keyword, indicating an increase in its attention.

Among them, Amino Acid Composition (1999, Intensity: 13.39, Time span: 1999–2018) has the longest time span, while Patterns (2000, Intensity: 32.04, Time span: 2000–2012) exhibits the highest intensity. Currently, research is more inclined toward Deep Learning (2018, Intensity: 28.69, Time span: 2020–2024), Mutations (2018, Intensity: 10.57, Time span: 2020–2024), and Artificial Intelligence (2019, Intensity: 23.33, Time span: 2021–2024). Early citation bursts (1999–2015) primarily focused on biological patterns, cancer proteomics, and early ML methods, whereas recent citation bursts (2020–2024) center on the application of deep learning, AI, and mutation analysis in proteomics.

## 4 Discussion

Recent advances in big data and ML have driven interdisciplinary integration in proteomics research. However, proteomics faces challenges like high-dimensional data, noise, and heterogeneity, which traditional methods struggle to address. ML, with its computational power and ability to extract and integrate features, offers efficient solutions, playing a growing role in life sciences and medicine. To the best of our knowledge, this is the first bibliometric study conducted in the field of ML and proteomics.

### 4.1 General information

To achieve a more comprehensive and intuitive analysis of this field, we conducted a literature search in the WoSCC for relevant studies published between 1997 and 2024, retrieving 5,134 research articles authored by over 20,000 researchers from more than 5,000 institutions. We explored the development trajectory, author collaboration networks, key publications, research hotspots, and emerging trends in this field. The findings offer new perspectives on the integration of ML and proteomics, deepening our understanding of this interdisciplinary domain and providing strong support for future research directions.

Data analysis shows a significant increase in academic output in the field of proteomics and ML from 1997 to 2024, with an average annual publication volume of 183 papers and an annual growth rate of approximately 45.14%. This trend reflects the rapid development of ML applications in proteomics, positioning it as a key direction in the intersection of life sciences and bioinformatics. Our findings support the application of Bradford’s law, a bibliometric concept suggested by Brookes (48).

The United States has published 1,289 papers, accounting for 25.06% of the total global output, ranking first globally. It also has the highest H-index (136), indicating the high academic impact of its research and reflecting its strong scientific capabilities and sustained research investments. The United States dominates the proteomics and ML research field, showing significant advantages in research quality, impact, and international collaboration. Not only that, its strong research infrastructure and high-impact contributions solidify its leading position in the field.

In contrast, while China has emerged as a significant contributor to global bioinformatics research—ranking as the second-largest producer with 1,106 papers—its performance remains comparatively limited in impact-based metrics, such as citation frequency and H-index (80). Although China’s rapid growth in research output is commendable, its centrality score (0.02) is exceptionally low, suggesting limited international collaboration. Indeed, one contributing factor, as highlighted in previous studies, is that Chinese researchers engage less frequently in cross-border scientific partnerships compared to their counterparts in Europe and North America. This relative isolation hinders the global dissemination and recognition of their work, ultimately diminishing citation impact and overall scientific influence.

International collaboration is crucial for enhancing research quality and academic impact, as demonstrated by the experiences of countries like the United Kingdom, Germany, and Australia. Furthermore, global research networks are increasingly coalescing into four major clusters, led by the United States, China, Europe, and Australia—a clustering trend that is expected to become even more pronounced in the future. Going forward, strengthening international collaboration, improving research quality, and promoting interdisciplinary integration will be key strategies for advancing this field and ensuring that burgeoning research outputs translate into greater global academic influence (49).

Further analysis was conducted to explore the relationship between national research strategies and scientific output (Supplementary Table 1). The analysis of major funding agencies reveals a strong alignment between national research priorities and publication output, suggesting that research funding plays a crucial role in driving outcomes that reflect strategic objectives. The United States dominates through health-focused investments, with the Department of Health and Human Services (HHS) and National Institutes of Health (NIH) collectively supporting 1,635 articles (including sub-agencies like NCI and NIGMS), reflecting its emphasis on biomedical innovation. In contrast, China’s National Natural Science Foundation (NSFC), the third-largest funder (689 articles), highlights its growing commitment to interdisciplinary science, though limited global collaborations (MCP%: 19.6%) suggest domestically driven initiatives. Europe leverages multinational coordination, with the European Union (EU) funding 267 articles alongside national agencies (e.g., Germany’s DFG, UK’s UKRI), collectively contributing 556 articles and exemplifying cross-border synergy. Notably, health-oriented agencies (e.g., NIH, NCI) dominate funding, while broader foundations (e.g., NSF, DFG) bridge computational and biological domains. These patterns mirror geopolitical research dynamics: United States -China competition in volume versus Europe’s collaborative model. However, hierarchical overlaps (e.g., NIH under HHS) necessitate caution in interpreting agency-specific contributions. Funding concentration in ML-proteomics underscores its strategic value for precision medicine, with disparities in international engagement shaping global knowledge networks.

An analysis of journal publication quantities helps to grasp the development trends of a discipline. It can identify the primary platforms for high-quality research publication, providing data support for optimizing research resources. This, in turn, aids research institutions and scholars in formulating more effective research strategies and collaboration plans, thereby promoting the continuous development of the proteomics and ML field. Studying the top journals in the field like Bioinformatics and Nature Communications can offer a good sense of where the research hotspots of the discipline lay, and current trends toward the future in these cross twain field. In addition to study quality, these indicators provide useful guidance to researchers, allowing them to determine which journals would contribute most significantly to the impact and visibility of their work.

To gain a comprehensive understanding of the foundational research and evolutionary trends within the field, we conducted a co-citation analysis of the literature from 1997 to 2024 (Supplementary Figure 1). The results reveal a clear temporal transition in research priorities. In the early 2000s, pioneering studies by Petricoin et al. and Adam et al. focused on applying mass spectrometry for tumor biomarker discovery, laying the groundwork for experimental platforms in proteomics (50, 51). During the 2010s, the maturation of ML methodologies—exemplified by the development of LIBSVM by Chang and Lin—facilitated the automated processing of high-throughput proteomic data (52). Entering the 2020s, deep learning techniques have been increasingly integrated into protein structure and function prediction, marking a new phase of rapid advancement. A landmark contribution by Jumper et al. introduced AlphaFold, which achieved a breakthrough in protein structure prediction and became a highly influential reference in the field (53). This line of research has also been closely linked with resources such as the STRING database developed by Szklarczyk et al., enabling the convergence of structural proteomics and network biology (54). Overall, the co-citation network highlights a paradigm shift from traditional experimental proteomics to AI-driven structural and functional prediction, underscoring the deepening integration of proteomics with artificial intelligence technologies.

Among the highly cited references identified in the burst detection analysis, the STRING database stands out as a particularly influential resource. The study by Szklarczyk et al., which introduced STRING v11, exhibited one of the strongest and most sustained citation bursts from 2020 to 2024. STRING provides a comprehensive platform for predicting and visualizing protein–protein interaction networks based on both experimental and computational data sources (54). In recent years, its utility has expanded significantly in proteomics research, particularly through its integration with systems biology and ML methods. By enabling high-confidence functional association networks, STRING has facilitated deeper insights into cellular mechanisms, disease pathways, and biomarker discovery, especially in complex disease contexts such as cancer and metabolic disorders (55).

The prominence of STRING in citation bursts aligns with our findings, which emphasize the increasing importance of integrative tools that combine proteomic data with ML techniques (56). Our study also highlights the critical role of network-based approaches in improving prediction accuracy and biological interpretability. STRING’s ability to contextualize omics data through curated interaction networks strongly supports the broader trend we observed toward multi-omics integration and the application of ML in precision medicine (57). Therefore, the elevated attention to STRING corroborates our conclusion that interaction-centered analysis frameworks are becoming central to advancing proteomics-driven biomedical research.

### 4.2 Author contributions

Using a review of the author’s works, we conclude that Professor Guo Tian-nan has been a leading contributor in integrating proteomics with ML, with a particular focus on the early diagnosis, personalized treatment, and prognosis prediction of malignant and metabolic diseases. His research has significantly advanced the application of ML techniques in clinical proteomics, especially in uncovering disease biomarkers and developing predictive models that support precision medicine (58, 59). He also has worked to develop methods to detect early-stage cancer biomarkers and optimize treatment strategies to improve personalized therapies by integrating ML techniques with proteomic data analysis (60, 61). In addition, Professor Guo has also promoted the integration of multi-omics data, including proteomics, metabolomics and genomics, to investigate molecular mechanisms of diseases, identify new biomarkers, etc., which provides theoretical basis for precision medicine (37). In cancer prognosis analysis, he has established proteomic-based survival prediction models that allow clinicians to better predict patients’ survival and recurrence risks (62, 63). As technology advances further, Professor Guo believed that his research results would play a greater clinical role in the identification, treatment, and prognosis management of more patients and provide new impetus for innovation in the biomedical field.

Professor Harald Mischak is widely recognized as a leading pioneer in clinical proteomics, having made groundbreaking contributions to the development and application of urinary proteomics for disease diagnosis (64), patient stratification, and prognostic assessment (65). His work has fundamentally advanced the field by establishing novel biomarkers and methodologies that have shaped current clinical practices. He was one of the pioneers in promoting the use of urinary biomarkers in chronic kidney disease and cardiovascular diseases (66, 67), and developed a series of non-invasive diagnostic methods based on proteomic fingerprinting (68). These tools have undergone rigorous validation in clinical trials and use for early detection of disease. In addition, he has pioneered the standardization of proteomic data and large-scale data integration that will better enable the use of data from across multiple centers and for use in translational medicine (69). Professor Mischak has also played a leading role in the clinical translation of biomarkers, facilitating the transition of urinary proteomics from laboratory research to real-world clinical applications, thereby providing a solid scientific foundation for precision medicine and personalized therapy (70).

Matthias Mann is widely regarded as a trailblazer in proteomics, having pioneered high-throughput mass spectrometry techniques that revolutionized protein identification and quantification. His innovative methodologies have significantly propelled the field forward, enabling more comprehensive and precise proteomic analyses (71, 72), such as shotgun proteomics and SILAC (Stable Isotope Labeling by Amino Acids in Cell Culture) (73, 74). These methods have significantly enhanced the depth and accuracy of protein detection, enabling precise measurement of dynamic protein changes within cells (75).

Furthermore, he played a crucial role in advancing ultra-high-resolution mass spectrometry, particularly Orbitrap technology, which has allowed researchers to analyze proteins and PTMs with unprecedented sensitivity and resolution (76, 77). His work has provided a strong technological foundation for large-scale proteomics research and has greatly expanded the application of proteomics in biomedical science, driving advancements in disease research, biomarker discovery, and precision medicine (78, 79).

### 4.3 Key research themes

Analysis of Keyword Clusters identifies and groups high-frequency keywords to reveal research hotspots, thematic evolution trends, and the relationships between different research directions. It helps explore the development trajectory of a discipline, analyze the emergence or decline of research topics, and quantify the knowledge structure of a specific field. The following are the six keyword clusters in this research field.

Proteomics research has undergone a significant transformation, evolving from database construction to ML integration, and more recently, to AI-driven precision medicine. Early studies primarily focused on protein databases (#4) and peptide identification (#3) (80). The establishment of databases such as Swiss-Prot and TrEMBL provided essential data support for protein classification, structural prediction, and functional annotation (81). Meanwhile, advancements in mass spectrometry significantly enhanced the accuracy and throughput of protein identification and quantification, enabling researchers to analyze complex biological systems and expand the applications of proteomics in disease research.

With the rapid accumulation of biomedical data, ML techniques, particularly Support Vector Machines (SVM, #2), were introduced into proteomics to facilitate protein classification, disease-associated protein screening, and biomarker discovery (82). ML approaches, including SVM, improved feature selection efficiency, allowing researchers to extract key patterns from vast proteomic datasets and develop predictive disease models. At the same time, serum proteomics (#1) gained increasing attention as a critical approach for biomarker discovery. The rich protein content in serum, when analyzed using MS combined with ML, greatly enhanced disease screening sensitivity and specificity, showing great potential in early diagnosis, particularly for cancer and neurodegenerative disorders (83, 84).

More recently, advancements in AI have propelled proteomics into the era of precision medicine, with a particular focus on Alzheimer’s disease (#0) and gene expression (#5) research (85, 86). AI-driven models have significantly improved the accuracy of disease risk prediction while promoting the integration of multi-omics data, including genomics, proteomics, and metabolomics. In Alzheimer’s research, AI is increasingly used to uncover relationships between proteins, gene expression, and clinical phenotypes, leading to the identification of more precise biomarkers for early diagnosis and therapeutic intervention (87–89). Similarly, gene expression studies have entered a new phase of multi-omics integration, where transcriptomic and proteomic data are analyzed in tandem to elucidate regulatory networks and molecular mechanisms underlying various diseases, ultimately paving the way for personalized treatment strategies.

Looking ahead, deep learning and multi-omics integration are expected to further enhance proteomics research, driving it toward greater intelligence and precision. The incorporation of deep learning will refine protein function prediction models, improve disease screening capabilities, and facilitate the development of personalized intervention strategies. As multi-omics data continues to be integrated.

### 4.4 Hotspots and trends

Highly cited literature analysis and keyword analysis are helpful to understand the development trends and hotspots of the interdisciplinary research field of ML and proteomic.

Since 2016, the application of AI and ML in proteomics has increased dramatically. Studies such as Bateman A, Demichev V, and Jumper J highlight the use of AI-driven protein structure prediction, protein interaction prediction, and deep learning techniques in the analysis of proteomic data (42, 44, 45). The citation bursts for these papers underscore the growing importance of AI methods, particularly AlphaFold, deep reinforcement learning, and generative adversarial networks (GANs), which are revolutionizing how biological data is processed. Research after 2020 demonstrates that AI and ML are increasingly being applied to enhance precision medicine and personalized treatment strategies. For example, studies by Szklarczyk D and Jumper J show that AI models are being used to deeply understand disease mechanisms, identify biomarkers, and predict protein functions with remarkable accuracy. The integration of proteomics, genomics, and AI is driving more precise therapeutic targeting, especially in the fields of cancer and neurodegenerative diseases (39, 45).

This transition highlights the shift in proteomics research from rule-based pattern recognition to intelligent algorithm-driven deep analysis. These newer techniques have addressed bottlenecks in data analysis, offering greater flexibility and innovation. As AI and deep learning continue to evolve, future research will increasingly focus on cross-disciplinary data integration and the development of refined disease models, paving the way for breakthroughs in precision medicine, early disease prediction, and drug discovery (90, 91).

#### 4.4.1 Deep learning

Recent literature highlights that deep learning has emerged as the most extensively utilized ML approach in medical research (92). Nonetheless, its implementation in certain domains—such as pneumonia diagnosis—remains relatively limited (93). Concurrently, a growing body of studies is investigating the potential of artificial intelligence, particularly deep learning, in the diagnosis of cytopathological conditions (94). These developments are consistent with our findings, suggesting that the future trajectory of deep learning in medicine will increasingly center on disease detection, characterization, and diagnostic enhancement.

Building upon this trajectory, deep learning has experienced a sustained citation surge from 2020 to 2024, underscoring its expanding influence within the field of proteomics (95). Deep learning algorithms, especially CNNs and Recurrent Neural Networks (RNNs), have made significant strides in protein structure prediction, functional annotation, and large-scale biomarker discovery (96). These techniques enable accurate modeling and analysis of complex biological data, unveiling protein structures and functional domains that were previously difficult to predict (97). Deep learning has also proven invaluable in drug target discovery and functional annotation, allowing researchers to extract meaningful insights from vast proteomic datasets, thus advancing personalized medicine (98, 99).

With the continuous development of deep learning technologies, its impact extends beyond proteomics, driving breakthroughs in computational biology, structural biology, and drug discovery (100). Deep learning is increasingly critical in precision medicine, helping identify disease mechanisms, discover biomarkers, and inform drug development. As computational power and algorithms evolve, deep learning will play a pivotal role in advancing medical applications, from diagnosis to therapeutic development (101).

#### 4.4.2 Mutations

From 2020 to 2024, mutations became a primary research focus. Researchers are transforming the understanding of how genetic mutations effect both structure and disease progression through ML model integration with genomic and proteomic data (102). This is especially important in the context of cancer genomics and neurodegenerative diseases in which specific mutations frequently act as an engine of tumorigenesis or neurodegeneration (103). In a recent study on cancer, it was found that most of the available tools were heterogeneous in terms of sensitivity and specificity. The study constructed a ML-based algorithm, DriverDetect, that combines the output of seven pre-existing tools to improve prediction of candidate driver cancer mutations (104). This is consistent with the view of this paper, with the advancement of algorithms and technology, the future development tends to be more toward federated learning and building a secure data sharing framework.

Cancer progressions are strongly associated with genetic mutations that alter the structure of the protein and change the results of function leading to the formation and development of tumors (105). Genomics and proteomics can be integrated to detect mutations associated with cancer for diagnosis and target therapy (106). A common type of mutation is activist mutation, which maintains the activation of a protein that works for tumor proliferation, such as KRAS gene mutation the other type is tumor suppressor mutation, such as TP53 mutation; the latter leads to the losing of function to suppress tumor proliferation (107). Mass spectrometry-based proteomics methods enable detection of alterations in protein levels in cancer cells, discovery of candidate biomarkers, and directed research on targeted therapy (108). Applying ML to large datasets of genomes and proteins, researchers can predict with unprecedented accuracy how mutations change the structure and function of proteins (102). Knowledge of mutation specific alterations in protein function within a framework of precision medicine enables tailoring therapies based on genomic information of the individual (102). Moreover, the combination of proteomics, genomics, and ML is pushing the algorithms that identify mutation-driven disease pathways (109), enabling mutation-based disease models to be generated more accurately. These developments not only improve our ability to predict disease outcome but also help to speed drug development strategies (110).

Due to the constant development of ML models, it is predicted that their capacity in unveiling intricate mutation-disease relationships will accelerate the shift from classical therapy to individualized medicine.

#### 4.4.3 Artificial intelligence

Since 2021, the citation burst of AI in proteomics has reflected its rapid development and expanding influence in the field (111). Such as in the areas of biomarker discovery, protein structure prediction, and disease diagnostics (112). And especially the combination of AI methods like NLP, deep reinforcement learning (113), and GANs is revolutionizing how biological data is processed, enhancing both the efficiency and accuracy of data analysis (114, 115). AI enables researchers to explore protein structures more thoroughly, discover proteins associated with diseases, and study protein-protein interaction networks, which helps to elucidate disease mechanisms and promotes the development of novel therapeutic methods (116).

AI’s role in proteomics is particularly evident in its ability to handle vast amounts of biological data and uncover complex patterns hidden within the data, overcoming the limitations of traditional experimental methods. For example, through deep learning models, AI can identify potential disease biomarkers within large-scale proteomic datasets, significantly accelerating early disease diagnosis (117). Furthermore, AI’s application in protein structure prediction, particularly through models like AlphaFold3 (118), has provided unprecedented accuracy in predicting protein 3D structures, which has profound implications for understanding protein function and drug design (119). However, there are still some problems in deep learning-based biological sequence representation, such as the lack of interpretability, which is currently one of the mainstream research directions of deep learning and is widely regarded as a key part of the next generation of AI technology (120). The application of proteomics is currently mainly focused on the discovery of biomarkers in serum or urine, and may tend to elucidate disease mechanisms and drug discovery in the future (121, 122).

As AI technology continues to evolve, the application of AI in proteomics will become more in-depth and promote the change of precision medicine and personal treatment strategies. AI can tailor treatment plans for individual patients based on their genomic and proteomic data, helping identify more effective targeted therapies, enhancing treatment efficacy, and reducing side effects. This will not only assist in the treatment of complex diseases such as cancer and neurodegenerative disorders but will also foster innovation in global medical research, helping address the current challenges in healthcare that remain difficult to overcome.

In summary, Early proteomics research centered on protein expression profiling, mass spectrometry optimization, and biomarker discovery, with keywords like protein expression, mass spectrometry, and biomarker discovery reflecting this experimental focus (Supplementary Figure 2). Around 2010, the increasing complexity of proteomic data spurred the adoption of ML, evidenced by terms like feature selection, SVM, and classification, signaling a shift toward data-driven approaches.

From 2015, deep learning further transformed the field, moving from tool-based analyses to integrated, systems-level strategies. Keywords such as neural networks, multi-omics integration, and pathway analysis became more prominent, indicating growing interest in biological interpretability.

In recent years, AI and big data advancements have ushered in a more “intelligent” era. The rise of terms like transformer models, self-supervised learning, and explainable AI reflects a focus on interpretability and clinical relevance. Meanwhile, applications such as precision medicine and spatial proteomics underscore proteomics’ expanding role in early diagnosis, personalized treatment, and intelligent healthcare.

Alongside this evolution, research trends indicate that proteomics is expanding beyond traditional biology into computational science, medicine, and environmental science, forming a highly interdisciplinary research framework that continues to evolve with advancements in ML and data-driven methodologies At the same time, proteomics research has extended beyond traditional biology into computational science, clinical medicine, and environmental science, forming an increasingly interdisciplinary framework.

### 4.5 Limitation

This study employs bibliometric methods to deliver the first comprehensive analysis of the current status and developmental trends in proteomics research utilizing ML techniques over the past two decades. Nonetheless, several inherent limitations should be acknowledged. Firstly, although the WoSCC is a widely recognized and authoritative database for bibliometric studies, our analysis was confined to this single source, which may limit the breadth of coverage and omit relevant publications indexed elsewhere. Second, only English-language literature was included, which may have led to the omission of relevant studies published in other languages.

## 5 Conclusion

The integration of ML into proteomics is currently advancing at a rapid pace, with current research hotspots centering on deep learning algorithms, the deployment of pre-trained models, and integrative multi-omics analyses. Looking ahead, future studies should prioritize the development of interpretable models with high clinical utility, foster interdisciplinary collaboration, and establish standardized, secure frameworks for data sharing. Moreover, leveraging longitudinal data for dynamic disease monitoring holds significant potential for advancing precision medicine. Collectively, these efforts are expected to promote broader data accessibility and collaboration, ultimately contributing to a more comprehensive systems-level understanding of complex diseases.

## Data Availability

The original contributions presented in the study are included in the article/Supplementary material, further inquiries can be directed to the corresponding authors.

## Supplementary material

Supplement Figure 1 Co-citation analysis of references.

Supplement Figure 2 Thematic evolution of machine learning in proteomics research across four time periods: **(A)** (1997–2005), **(B)** (2005–2015), **(C)** (2015–2022), and **(D)** (2022–2024).
