# Supplementary material for: Progress and trends on machine learning in proteomics during 1997-2024: a bibliometric analysis
Source: Front Med (Lausanne). 2025 Aug 15;12:1594442. doi: 10.3389/fmed.2025.1594442 (PMC12401104; doi:10.3389/fmed.2025.1594442)
Supplement: Supplementary file 1 [file Table_1.docx]

| Rank | Funding Agencies | Country | Articles |
| --- | --- | --- | --- |
| 1 | United States Department of Health Human Services | USA | 819 |
| 2 | National Institutes of Health NIH USA | USA | 816 |
| 3 | National Natural Science Foundation of China NSFC | China | 689 |
| 4 | European Union EU | EU | 267 |
| 5 | UK Research Innovation UKRI | UK | 168 |
| 6 | National Science Foundation NSF | USA | 167 |
| 7 | NIH National Institute of General Medical Sciences NIGMS | USA | 135 |
| 8 | German Research Foundation DFG | Germany | 121 |
| 9 | NIH National Cancer Institute NCI | USA | 121 |
| 10 | Spanish Government | Spain | 110 |

Supplement Table 1. Top 10 Funding Agencies
