# Supplementary figures and images for: Progress and trends on machine learning in proteomics during 1997-2024: a bibliometric analysis
Source: Front Med (Lausanne). 2025 Aug 15;12:1594442. doi: 10.3389/fmed.2025.1594442 (PMC12401104; doi:10.3389/fmed.2025.1594442)

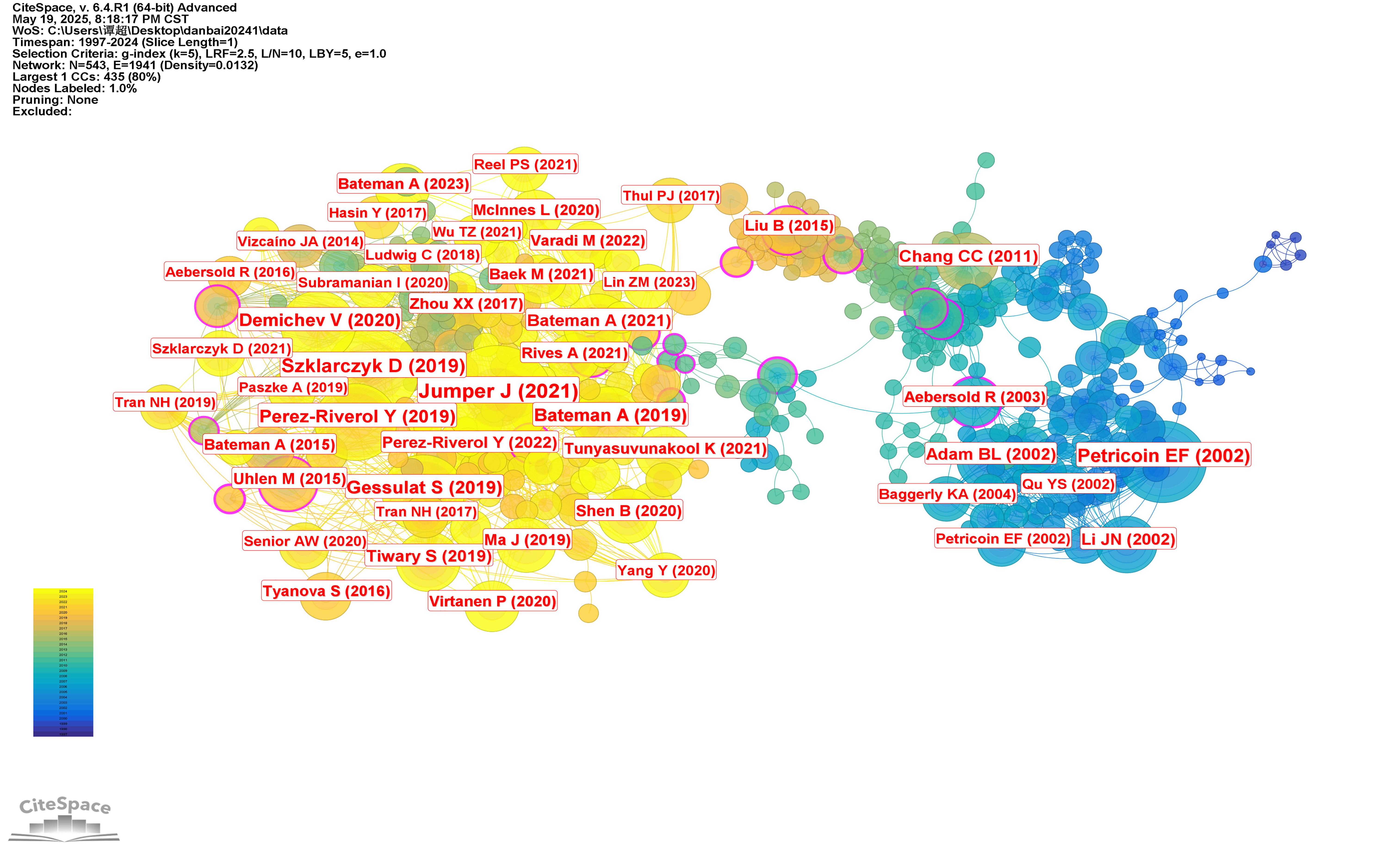

Supplement: Supplement Figure 1 — Co-citation analysis of references. [file Image_1.png]

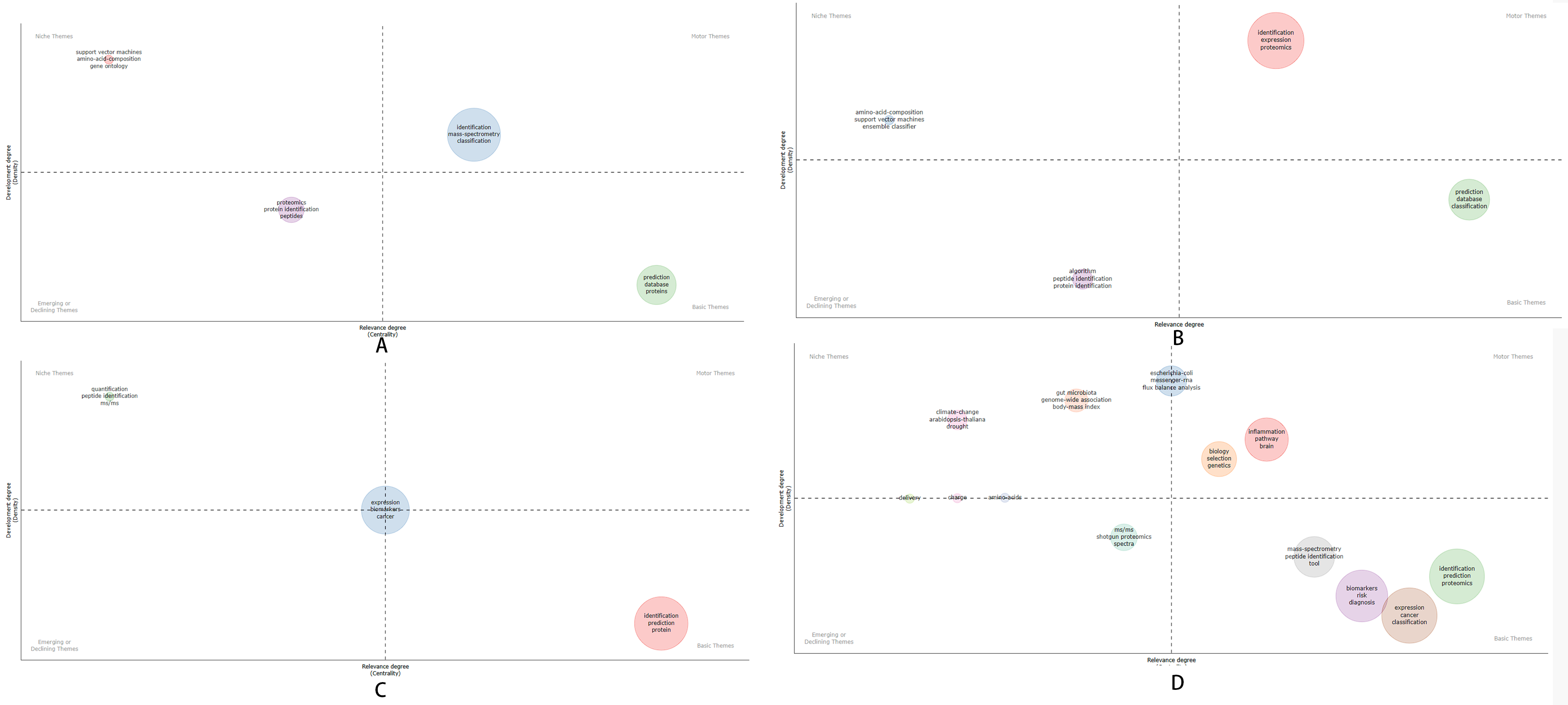

Supplement: Supplement Figure 2 — Thematic evolution of machine learning in proteomics research across four time periods: (A) (1997–2005), (B) (2005–2015), (C) (2015–2022), and (D) (2022–2024). [file Image_2.tif]
